# Strategies for Controlling Non-Transmissible Infection Outbreaks Using a Large Human Movement Data Set

**DOI:** 10.1371/journal.pcbi.1003809

**Published:** 2014-09-11

**Authors:** Penelope A. Hancock, Yasmin Rehman, Ian M. Hall, Obaghe Edeghere, Leon Danon, Thomas A. House, Matthew J. Keeling

**Affiliations:** 1 Department of Zoology, University of Oxford, Oxford, United Kingdom; 2 School of Life Sciences, University of Warwick, Coventry, United Kingdom; 3 Field Epidemiology Service (Birmingham), Public Health England, West Midlands, United Kingdom; 4 Emergency Response Unit, Public Health England, Porton Down, United Kingdom; 5 School of Mathematics, Queen Mary University of London, London, United Kingdom; 6 Warwick Mathematics Institute, University of Warwick, Coventry, United Kingdom; The Pennsylvania State University, United States of America

## Abstract

Prediction and control of the spread of infectious disease in human populations benefits greatly from our growing capacity to quantify human movement behavior. Here we develop a mathematical model for non-transmissible infections contracted from a localized environmental source, informed by a detailed description of movement patterns of the population of Great Britain. The model is applied to outbreaks of Legionnaires' disease, a potentially life-threatening form of pneumonia caused by the bacteria *Legionella pneumophilia.* We use case-report data from three recent outbreaks that have occurred in Great Britain where the source has already been identified by public health agencies. We first demonstrate that the amount of individual-level heterogeneity incorporated in the movement data greatly influences our ability to predict the source location. The most accurate predictions were obtained using reported travel histories to describe movements of infected individuals, but using detailed simulation models to estimate movement patterns offers an effective fast alternative. Secondly, once the source is identified, we show that our model can be used to accurately determine the population likely to have been exposed to the pathogen, and hence predict the residential locations of infected individuals. The results give rise to an effective control strategy that can be implemented rapidly in response to an outbreak.

## Author Summary

Public health strategies for infectious disease control can benefit greatly from our growing capacity to predict human movement behaviour. This is facilitated by modern methods of electronic data generation and storage that allow us to track detailed human movement patterns. Here we develop a mathematical model of the dynamics of non-transmissible infections that is informed by a new data set describing detailed movements of the population of Great Britain. We apply the model to three outbreaks of Legionnaires' disease. We demonstrate how the method can assist during the crucial early stages of an outbreak by providing predictions of the infection source location and individuals with a high exposure risk.

## Introduction

The development of epidemiological models to inform public health strategies for infectious disease control has been greatly aided by incorporating an understanding of human movement behaviour, drawing on the increasing availability of data describing human movement patterns [1]–[5]. Recent studies emphasise the need to include information on a range of movement activities in addition to home-workplace commuting, such as irregular and stochastic movements, in order to accurately predict important properties of epidemics such as the rate of spatial spread of transmissible infections [6] and the location of sources of non-transmissible infections [4]. Models informed by detailed data describing a range of movement activities, such as mobile phone data and simulated traffic flow data, can then be used to develop targeted intervention strategies, for example vaccinating high risk individuals [2] and increasing surveillance on high risk travel routes [5].

In this study we utilize a source of human movement data that has not previously been applied to inform infectious disease control: a high-resolution database developed within the retail sector that describes travel behaviour for work, shopping and educational activities by the population of Great Britain. This database, which we term the Great Britain Human Movement (GBHM) database, has been developed by a commercial retail planning consultancy in order to forecast the sales potential of retail development sites. Location-specific estimates of consumer demand are generated using fine-scale predictions of population movements to parameterise a spatial interaction model [7], [8] (see Section S9 of Text S2). The model is informed by socio-demographic and travel data from a range of sources including publicly available data from the United Kingdom census [9] as well as commercial information on customer travel and demography collected from store loyalty cards and electronic point-of-sale records [7], [10] (see Sections S1 and S9 of Text S1 and Text S2).

Infectious diseases that are never or very rarely transmitted between humans, including *Legionella pneumophilia* (Legionnaires' disease), H5N1 (avian flu), and inhalational anthrax, are typically contracted from a localized infection source. When an outbreak is detected the primary public health concerns are to locate (and treat) the source of infection and rapidly identify the individuals who are likely to have been exposed; these combined actions aim to prevent further infections and enable early treatment of affected individuals [4]. Both of these objectives require a detailed understanding of the population's movements. A location's potential for being the infection source is influenced by the total number of infected and uninfected individuals that visited the location. Additionally, an understanding of population movements can help to identify high risk groups and hence target surveillance for undetected infections [4].

Here we develop a mathematical model for the dynamics of non-transmissible infections that is informed by the commercial GBHM database. We explore the model's ability to inform the response to outbreaks of Legionnaires' disease, a potentially life-threatening form of pneumonia that is contracted when a susceptible human inhales aerosolized water containing the bacteria *Legionella pneumophilia* [11]. There have been many community-acquired *Legionella* outbreaks associated with environmental sources including cooling towers [12]–[16], whirlpool spas [17], [18] and supermarket mist machines [19]. Locating such infection sources is often difficult and time-consuming for public health workers [20], [21].

We use the model to predict the location of the infection source and the individuals in the population with a high risk of exposure for three outbreaks of Legionnaires' disease that have occurred in Great Britain. Our analysis asks whether predictive capacity is improved by increasing the detail of the data describing population movement patterns. We find that the most accurate prediction of the source location is obtained when individual-level information about the travel histories of infected individuals is used to inform the model. However, given the debilitating effect of Legionnaires' disease, obtaining movement histories from infected individuals is time-consuming and sometimes impossible; we therefore need to consider alternative sources of movement data [17], [22]. Using the GBHM database to estimate movement probabilities still predicts the source location to within a narrow local area. Moreover, the home locations of high-risk individuals are predicted with high accuracy using this movement database. When simple dispersal kernels are used instead of these more detailed movement estimates, the predictive accuracy and confidence declines significantly. This suggests that relating human movement patterns to the particular urban geography of the study region is important to shaping our predictions.

## Methods

### Fine scale spatial movement patterns of the population of Great Britain

Predictions of the spatial movement patterns of all individuals in England, Scotland and Wales are provided by a large data set (the GBHM database). These movement predictions are informed by commercial and public data describing a range of individual movement activities including commuting between home and workplace, shopping trips and visits to schools and higher education institutions. A detailed description of the data sources and prediction methodology is given in sections S1 and S9 of Text S1 and Text S2 while the main features of the data are described here.

**Figure 1:**
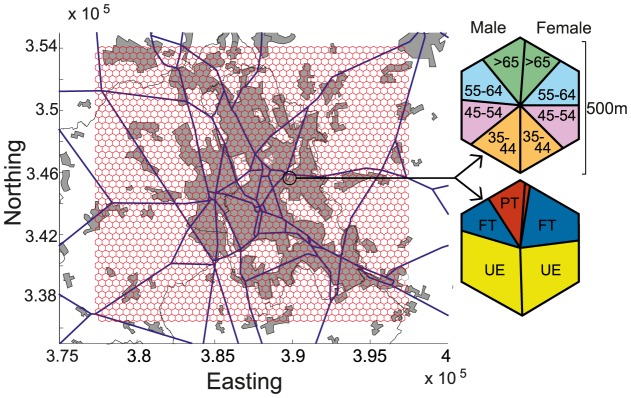
A hexagonal mesh showing the spatial units of the Great Britain human movement database. A subset of the data covering the city of Stoke-on-Trent is shown. For an example hexagon magnified on the right, coloured segments correspond to the proportion of the residents in different demographic categories, considering only individuals older than 34 years of age. The top hexagon shows age ranges in years and the bottom hexagon shows employment status (UE  =  unemployed, FT  =  full-time employed and PT  =  part-time employed). The gray shaded areas on the map indicate urban areas and the blue lines indicate major roads.

The total area of England, Scotland and Wales is subdivided into hexagonal spatial units of 500 m in diameter, resulting in approximately 21 million spatial units. The database contains estimates of the number of individuals residing in each hexagon (based on census data) stratified by socio-demographic variables including age, gender and employment status (full-time employed, part-time employed, unemployed, economically inactive or full-time education) (Figure 1). For an individual with a given residence hexagon and socio-demographic type, the database provides estimates of the probabilities of visiting all locations in the landscape; these visits are subdivided by activity type: work, education at schools or universities, shopping for food and non-food consumables and other unknown activities. The probabilities also depend on the time and day of the week in which the activity is undertaken. The week is divided into 28 components, four components for each day, which are defined as night (8pm–6am), peak morning (6am – 10am), day (10am–4pm) and peak evening (4pm–8pm) (see section S1 of Text S1).

The data show that spatial movement patterns vary considerably with the reason for travel. Shopping destinations receive high rates of visitation because shopping activity is distributed across a relatively small number of destinations, with individuals preferring to shop close to home. In contrast, work activity is distributed across a larger number of destinations and so visitation to these locations is generally less intense (see section S1 of Text S1).

### Case data for outbreaks of Legionnaires' disease in Great Britain

Data describing three outbreaks of Legionnaires' disease in different areas of England (Stoke-on-Trent, Hereford and Barrow-in-Furness) were used to assess the performance of our methodology and the impact of movement data quality. For each of the outbreaks the source of infection and its location have been identified by traditional field epidemiology and confirmed by laboratory studies.

The Stoke-on-Trent outbreak consists of 23 laboratory-confirmed cases of Legionnaires' disease identified between May and August 2012 [17]; age, gender, place of residence, occupation and date of symptom onset were recorded for all cases. In addition, the Health Protection Agency in the West Midlands conducted repeated interviews of each case (or sometimes their relatives) to obtain detailed travel histories over a period of 2 weeks prior to the date of symptom onset. This time period encompasses the bulk of the estimated maximum incubation period of *Legionella pneumophilia* (<10 days in ∼90% of cases) [18], [20] (see section S1.3 of Text S1).

The outbreak in the city of Hereford has 28 cases that were identified between October to November 2003 [13]. The data provide demographic information on only 19 of the cases including age, gender, place of residence and occupation. Travel history data for the cases is not available.

The Barrow-in-Furness outbreak was the largest with 179 confirmed cases reported in August 2002 [16]. For this outbreak the data provide the residential locations of 96 of the cases but does not include any information about the demography or travel history of the cases.

### Modelling non-transmissible infection dynamics

We define a region *R* surrounding the outbreak area within which each hexagonal unit could possibly contain the source of infection. We assume that the infection rate within the source hexagon, 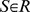, is a constant value, 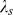. Further, individuals who reside in hexagon *S* are assumed to experience a different infection risk (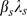 where 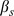 is a constant) when they are at home compared to individuals visiting the location. For every individual *i* in the population the probability of becoming infected by a source located within hexagon *S* is 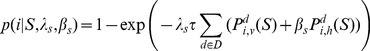
(1)


where 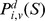 is the probability that individual *i* is present in location *S* on day part *d* and not at home and 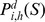 is the probability that individual *i* is at home in location *S* on day part *d. D* is the period over which the individuals are exposed to a risk of infection and 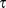 is the duration of a day part, which is approximated to one-quarter of a day. The values of 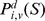 and 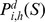 can either be estimated from recorded travel-history patterns (if such information is available), estimated from the GBHM data-base, or approximated using household location data and a simple movement kernel. (A derivation of (1) and the associated probabilities is given in sections S1 and S2 of Text S1).

We now use the infection probabilities (1) to determine the likelihood that individuals in set *I* become infected while the rest (set *U*) remain uninfected over a time period *T_c_*:
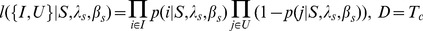
(2)


### Predicting the location of the infection source

To predict the location of the source of *Legionella pneumophilia* infection we first determine the maximum likelihood values of the infection rates 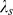 and 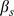 for each of the possible source hexagons 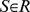. This is achieved using an open-source Bayesian Markov Chain Monte Carlo (MCMC) Gibbs sampling algorithm [23] to estimate the marginal posterior distribution 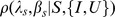 for each fixed value of *S*, using the above likelihood expression (eqn 2). Uniform priors 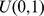 are used for the parameters 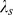 and 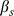 to ensure non-negative estimates. With this choice of prior distributions the mode of the marginal posterior 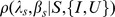 approximates the maximum likelihood value [24] (see section S2 of the Text S1).

We then rank the set of possible source hexagons in order of decreasing values of the Deviance Information Criterion (DIC) [24], [25]. We select a hexagon, *S*, as the preferred source location if the DIC value obtained from the estimate of 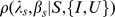 is lower by at least 3 compared to the DIC values for all other source locations [25]. We chose to predict the source location based on the DIC in order to account for differences in the effective number of parameters of the fitted models.

To assess how more detailed information on population movement patterns improves prediction of the infection source location, we considered three different levels of movement data richness, referred to as Levels 1, 2 and 3.

Level 1 is the most detailed and uses travel histories of cases to estimate their movement probabilities. However comprehensive travel histories are time- consuming to obtain especially as most individuals infected with Legionella are in a state of poor health. Repeated interviews of the cases and their relatives are often required [17], [22]. Therefore, we considered a subsample of the full set of travel histories to approximate the amount and quality of data that is likely to be available during an outbreak. Specifically, we considered only the first half of the total set of cases to develop symptoms, and of these we randomly selected only 50% of the destinations recorded for each case. Movement probabilities for uninfected individuals are constructed as in Level 2, below. This first level of data richness is only available for the Stoke-on-Trent outbreak.

Level 2 assumes that travel histories are not available, which represents the situation that public health agencies face in the early stages of an outbreak. The GBHM database was used to estimate the probabilities that all individuals, both infected and uninfected, visited location *S*. Due to the greater incidence of Legionnaires' disease in older individuals [11], [12], [26], only people of over 34 years of age are considered in the uninfected population.

Level 3 uses the least amount of individual-level movement information; the movement probabilities of infected individuals were estimated by a single power-law function of distance from home (see section S3 of Text S1). Such power-law kernels have been shown to accurately represent the distribution of workplace commuting journeys [3], but will not reflect attractiveness of particular locations or individual-level heterogeneities. The parameters of the power-law distribution were estimated using the GBHM database (see sections S2 & S3 of Text S1).

### Predicting the population at risk of infection

Once a location has been identified as a potential source of infection our methodology can be used to predict the risk of infection to all individuals in the population. Identifying the home locations of high-risk exposed individuals can help design intervention strategies that target these individuals [22] and provides an additional verification of our methodology. Using the GBHM database and the known source of infection two quantities of interest are calculated: the probability of each hexagon containing a case and the expected number of cases living within a given distance from the source.

We calculated the predicted probability of obtaining one or more infected individuals residing within a given hexagon, *H*:
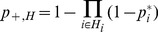
(3)


where *H_i_* is the set of individuals residing in hexagon *H* and 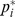 is probability of infection for individual *i* given by the mode of the posterior distribution for the model corresponding to the true source hexagon (see section S2 of Text S1).

Naturally, the probabilities 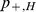 depend strongly on the distance of the residential hexagon from the source due to the strong distance-dependent nature of travel (see sections S1 and S3 of Text S1). We therefore compare values of 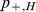 for the observed case home hexagons to the values for other hexagons a similar distance from the source. If the GBHM database accurately represents the subset of individuals most likely to visit a destination (beyond simple distance-dependence) then hexagons that contain home locations of the true/reported cases will have 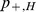 values that are above average for their given distance.

To further assess the model's prediction of the spatial prevalence we calculated the cumulative number of cases expected to live within a distance *r* from the source hexagon, 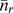, as
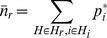
(4)


where *H_r_* is the set of all hexagons within a distance *r* from the source. The observed number of residential hexagons of cases within a given distance can then be compared to the expected value 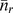.

## Results

### Predicting the location of the source of infection

We assess the ability of our methodology to predict the infection source location, considering the three outbreaks and three different levels of richness of the data describing human movement patterns. For each outbreak and data richness level, we compare values of the Deviance Information Criterion (DIC) for each hexagon that could possibly contain the source of infection. We will focus on the Stoke-on-Trent outbreak, for which we have more detailed information, but consider the Hereford and Barrow-in-Furness outbreaks with the same framework. Examples of the results of the MCMC algorithm for all of the analyses presented in this study are provided in section S4 and Table S5.1 in Text S1.

### The Stoke-on-Trent outbreak: Level 1

**Figure 2:**
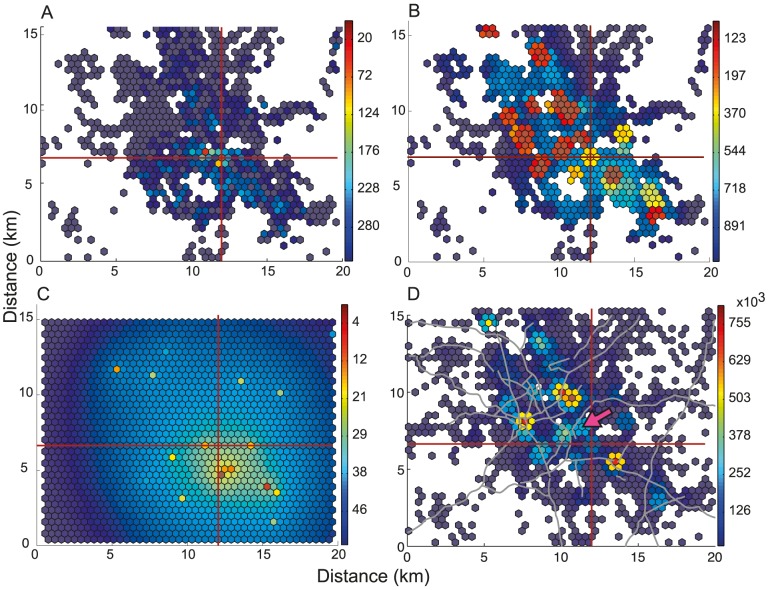
A–C. The deviation in DIC for different locations of the infection source for the Stoke-on-Trent outbreak. The three levels of richness of the human movement data are shown are shown: A. Level 1, B. Level 2, C. Level 3. The deviation in DIC is calculated as the difference from the lowest DIC value for the given data richness level. The red lines intersect at the location of the true infection source. Town features are not shown to preserve anonymity. **D.** The number of individuals with a non-zero probability of visiting locations in Stoke-on Trent during a week, predicted by the GBHM database. The pink arrow indicates the preferred source hexagon obtained using the GBHM database to describe human movement (data richness Level 2). Grey lines indicate major roads.

**Figure 3:**
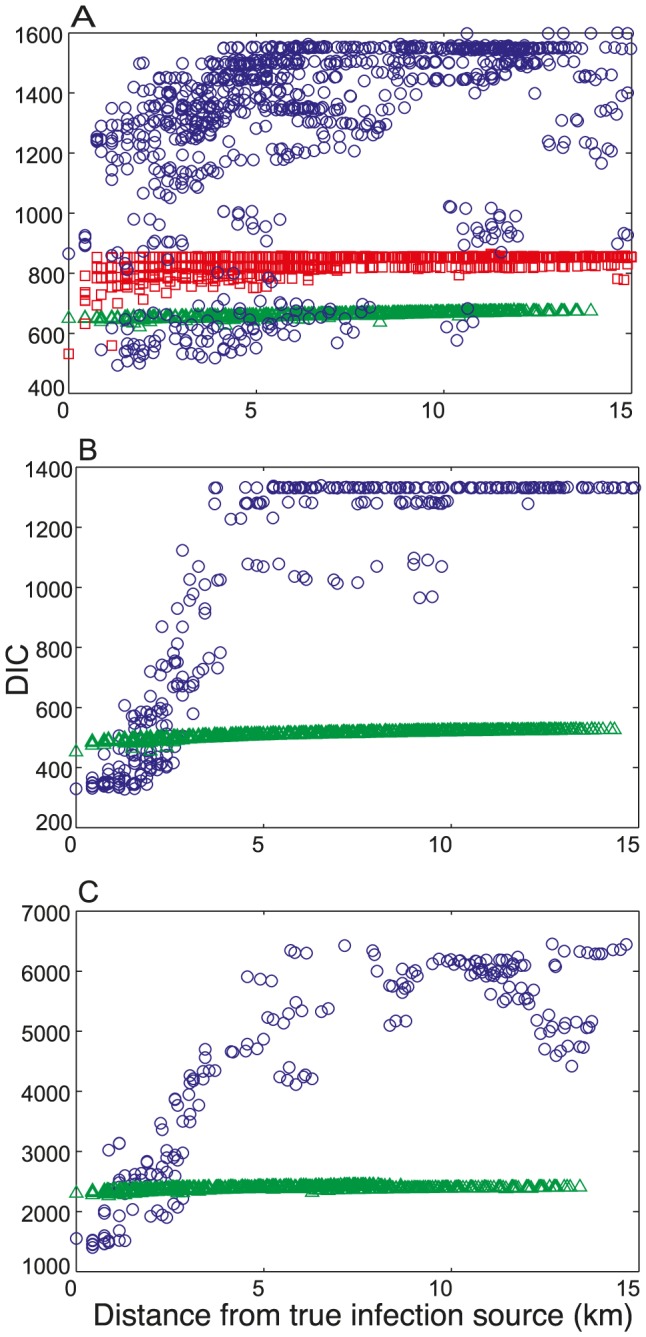
The DIC value for different locations of the infection source for the outbreaks in A. Stoke-on-Trent, B. Hereford and C. Barrow-in-Furness. The three levels of richness of the human movement data are shown: Level 1 (red squares), Level 2 (blue circles) and Level 3 (green triangles).

Using the recorded travel history information (richness Level 1), our method correctly identifies the hexagon containing the true source (Figure 2A). This hexagon is clearly preferred with a DIC value that is 27.6 and 99.9 lower than the second and third preferred source locations respectively (Figure 3A; red squares). Hence our method confidently and accurately predicts the infection source location when it is informed by travel history data, even though we only use the first 12 cases and randomly exclude half of all reported destinations in their travel histories.

### The Stoke-on-Trent outbreak: Level 2

Using the Great Britain Human Movement (GBHM) database (richness Level 2), the preferred hexagon is only 1 km away from the true source (Figure 2B). The differences between the DIC value of the preferred hexagon and the second and third ranked models are less than those obtained for Level 1 although there is still a clear preference (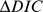 =  6.7 and 13.7 respectively; Table S5.1). Hexagons predicted likely to contain sources of infection occur in clusters that are often positioned on major shopping locations. The true source is the centre of such a cluster; however its DIC is relatively high as it is a less popular shopping destination with other more popular ones nearby (Figures 2B,D). The GBHM database predicts that 16 out of the 23 cases have a non-zero probability of visiting the source hexagon, in contrast the most preferred hexagons have a non-zero probability for all of the cases.

The results obtained for data richness Levels 1 and 2 demonstrate that travel histories from infected individuals are key to pinpointing the exact infection source location for the Stoke-on-Trent outbreak. This is because, even if the GBHM database perfectly captures the set of all likely movements (and assigns them a probability), the travel histories of cases eliminates particular locations while providing definite knowledge of movement to others - they provide a realization from the probability distribution. It is this categorical information that allows the source of infection to be accurately located using the travel-history data. Moreover, even partial travel-history data can be sufficient to determine the source location.

The GBHM database demonstrates that both the spatial location and the attractiveness of a destination are important factors in predicting the source location. Figure 3A (blue circles) shows that there is a subset of locations with substantially lower DIC values than the majority; these locations are destinations with strong attractiveness (Figure 2D). Within this subset of attractive destinations the models show an overall trend of preferring locations closer to the true infection source (Figure 3A). The map of the attractiveness of the destinations represented in the GBHM database (Figure 2D) shows that the top preferred source hexagon (indicated by the pink arrow) is not the most attractive destination in the area, but it is both close to the true source and more attractive than the other destinations that are closer to the true source.

Taken together, Figures 2 and 3A show that using the GBHM database enabled prediction of the infection source location to within a local area and also identified a ranked set of plausible candidate destinations based on their attractiveness to the infected individuals and the wider population. It therefore provides a useful alternative in the early stages of an outbreak when comprehensive travel history information is not available (see also the Discussion).

### The Stoke-on-Trent outbreak: Level 3

Assuming an isotropic, homogeneous, power-law movement kernel for all individuals (richness Level 3) leads to strong preference for home locations due to the localization of the movement kernel. The preferred source hexagon (Figure 2C) contains the home locations of two cases and is approximately 2 km away from the true infection source. This hexagon is clearly preferred compared to the second and third ranked models (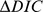 =  5.8 and 12.7 respectively; Table S5.1) which are also home locations of cases (see also the distribution of the residential locations in Figure S6.1 of Text S1). Notably, the DIC values using the movement kernel are far more uniform across all possible source locations compared to those obtained using richer movement data (Figure 3A), and our method does not provide a clear discrimination of the likely locations of the infection source apart from the cases' home locations.

### The outbreaks in Hereford and Barrow-in-Furness

For the outbreaks in Hereford and Barrow-in-Furness travel history data were not available, therefore only data richness Levels 2 and 3 were considered. For both of these outbreaks the urban area under consideration is relatively small compared to that of Stoke-on-Trent (see Figure S6.1 of Text S1) which allows more accurate prediction of the source location.

For the Hereford outbreak and data richness Level 2 the three most preferred hexagons are indistinguishable with DIC values of 328.0, 329.3 and 329.9 respectively (Table S5.1). The true source of infection corresponds to second lowest DIC value (see the map in Figure S7.1A of Text S1). For data richness Level 3 the two most preferred models are indistinguishable (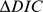  =  2.9), and true source of infection is associated with the lowest DIC value (see Figure S7.2A of Text S1). These results are attributable to the close clustering of case home locations, with 3 out of 19 of the cases residing in the hexagon that contained the infection source (see Figure S6.1 of Text S1). This means that infection in the home is more likely and hence 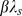 is substantially greater than zero (see Figure S4.1D of Text S1). Although both levels of data richness allow the source location to be accurately predicted, the GBHM database again provides greater discrimination between the candidate locations, with a clear trend preferring locations closer to the true source (Figure 3B).

For the Barrow-in-Furness outbreak and data richness Level 2, the preferred source location corresponds to a hexagon that is adjacent to the true infection source (see the map in Figures S7.1B). This hexagon is clearly preferred with a DIC that is lower by 47.4 and 57.4 compared to the second and third most preferred models (Table S5.1 of Text S1). For data richness Level 3 the preferred source location is about 1 km away from the hexagon containing the true source, and this hexagon is clearly preferred, with a DIC that is lower by 16.3 and 17.5 compared to those for the second and third most preferred hexagons respectively (see Figure S7.2B and Table S5.1 of Text S1). The source of this outbreak is part of a shopping destination in the centre of town; such locations are predicted by the GBHM database to be highly attractive with several cases travelling considerable distances from home to the source (see Figure S6.1 of Text S1). This explains both the low estimate of 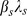 (even though 3 out of 96 of the cases resided in the source hexagon, see Figure S4.1C,E of Text S1) and the greater accuracy gained using data richness Level 2 (Figure 3C). Again there is less discrimination between the models for data richness Level 3 (Figure 3C).

### Predicting the population at risk of infection

**Figure 4:**
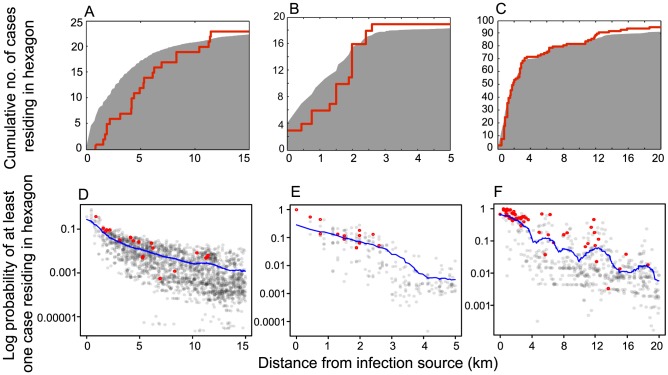
The cumulative number of residential locations of infected individuals (A–C), and the predicted probability of at least one infected individual residing in a hexagon (D–F), as a function of distance from the infection source. Columns of panels correspond to each of the three outbreaks: the Stoke-on-Trent outbreak (A,D), the Hereford outbreak (B,E) and the Barrow-in-Furness outbreak (C,F). In A–C the shaded grey area shows the expected cumulative number of cases and the red line shows the observed cumulative number of cases. In D–F the grey circles show the predicted probability values 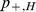 for all hexagons and the red circles show these probability values for the observed residential hexagons of cases. The blue line shows the moving average of the predicted probabilities 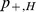
with a window of 2 km. In the case of the Barrow-in-Furness outbreak, one case resided 40km from the source and is not shown.

We now use the model informed by the GBHM database to predict the cumulative number of home locations containing cases within a given distance from the infection source (eqn 4). These predictions agree well with the observed data for all three outbreaks, providing good estimates of both the local gradient and eventual asymptote (Figure 4A–C). The distance range plotted differs for each outbreak, reflecting the variation in the scale of human movement patterns associated with the different urban geographies of each outbreak location. For example the distribution for the Barrow-in-Furness outbreak (Figure 4C) features infections at longer distances from the source because in this relatively low-density rural environment some individuals necessarily travel further distances to reach urban centres (see Figure S6.1 of Text S1). These long-range effects are well captured by the model and this data richness level (Figure 4C). In contrast, for the Stoke-on-Trent outbreak (Figure 4A) the model tends to over-estimate the proportion of the cases that lived close to the infection source. This can be explained by the highly contained dispersal of the *Legionella pneumophilia* associated with this outbreak (due to the source being housed indoors) [17], so that only a small proportion of individuals who visited the source hexagon were actually exposed to the infection source.

For a given distance from the source, the model-predicted probability that a hexagon contains a case, 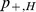, provides a more detailed assessment of our ability to capture spatial structure. Values of 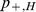 are relatively high for those hexagons that are observed case home locations (Figure 4D–F), indicating that the model provides a fine scale identification of areas where infected individuals are likely to reside, as well as the radial pattern about the source (see also Figure S8.1 of Text S1).

For the three outbreaks (Stoke-on-Trent, Hereford and Barrow-in-Furness) the probability 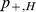 is greater than expected in 16 out of 20 (80%, *p*<0.02), 9 out of 13 (69%, *p*≈0.29) and 48 out of 56 (86%, *p*<10^−6^) case home locations respectively. The accuracy of these predictions relies on the location-specific nature of individual movement patterns that are informed by the urban geography of the outbreak region. This indicates that a detailed knowledge of the human movement landscape in the region surrounding an infection source can be of substantial benefit to infectious disease control by assisting in active case finding [22] and predicting the size and spatial extent of an outbreak.

## Discussion

We have assessed the ability of models informed by detailed human movement data to predict important spatial features of non-transmissible infections, focusing on *Legionella pneumophilia* outbreaks. Our analysis compares the predictive capacity afforded by detailed travel histories from infected individuals with that given by estimated movement patterns produced using simulation models that consider varying levels of movement complexity. Public-health management can benefit greatly from predictive tools that can be implemented prior to obtaining travel history data, which typically requires repeated interviewing of infected individuals, or often their relatives, depending on the individual's state of health [17], [22]. Epidemic curves for Legionnaires' disease outbreaks show that the majority of infections can occur in less than a week in some instances [12], [27], so rapid response informed by prompt analysis can be crucial.

The computational methods developed here provide a clear strategy to help reduce the incidence of infection in the early stages of a non-transmissible infection outbreak. Our database describing population movement patterns for Great Britain can be used to predict the local area containing the infection source and also identify plausible candidate source locations. In addition, the method can be used to predict the number and spatial distribution of future cases, giving public health organisations an advanced warning of the spatial extent of an outbreak, as well as a narrower target for more costly, detailed data collection. Our method can be applied iteratively to support an outbreak investigation by combining predicted travel patterns of the wider population with travel history information for infected individuals as this data becomes available. The resulting geographically detailed prediction of the infection landscape can be used as a visual tool to focus case and source finding activities on likely areas of high infection risk. This can help to make efficient use of the limited resources available to outbreak control teams.

Using the GBHM database (under the Level 2 analysis), our method provides a spatially precise prediction of the infection risk landscape because it is based on a fine scale identification of attractive and unattractive points in the landscape e.g. shopping centres versus empty fields. The method essentially ranks these locations by the likelihood that they contain the infection source based on predicted travel patterns of the infected individuals and the wider population. We have found that the top preferred source location is typically a relatively attractive site. Generally this predicted location does not exactly match the true source location, but it is within about 1 km from the true source for the three outbreaks analysed.

In contrast, when our method uses isotropic movement kernels centred on the case home locations to predict the movements of infected individuals (the Level 3 analysis), we essentially obtain a ranking of these home location neighbourhoods by their likelihood of being an infection source. The home locations of cases, and their surrounds, are obvious and sensible places for public health workers to look for infection sources. We believe that the GBHM database adds valuable information to predicting the infection risk landscape by identifying candidate source locations that are likely common sources of attraction to all of the cases, and are therefore also likely sources of infection. By predicting short range as well as long range journeys to particular attractive centres, the database is able to accurately predict infection risk over a large spatial area (Figure 4).

Our analysis has adopted a simple representation of the process of pathogen dispersal and dissemination, assuming that the infection is localized within the source hexagon and that the infection rate is constant across the entire period. The modelling study by Egan et al. [20] concludes that the assumption of a constant infection rate is appropriate for several *L. pneumophilia* outbreaks, although the true form of the variation clearly depends on the infection source and may also depend on weather conditions [21], [28]. Our method could be extended to consider more extensive pathogen dispersal at the cost of added computational expense; this may be usefully applied to analyse Legionella dispersal from cooling towers, which can potentially disperse the pathogen up to 7 km [14]. Detailed models of pathogen dispersal have been developed for *L. pneumophilia* [29], [30] and other non-transmissible pathogens [4], [31], but frequently rely on complex dispersion modelling influenced by local meteorological conditions.

Our model also simplifies the variation in susceptibility to *L. pneumophilia* infection that exists within the human population, assuming that susceptibility is constant and confined to older age classes. Susceptibility is known to increase with age and to be higher in males, particularly smokers [11], [12], [26]. However, there is currently a lack of knowledge regarding how infection risk depends on the inhaled dose of the pathogen [20], [22]. This limits our ability to quantify the variation in susceptibility within the population.

In conclusion we have demonstrated that the detailed understanding of human movement patterns and their interaction with the urban landscape, that have been developed largely for commercial reasons, can be successfully applied to prediction of non-transmissible infections. Representing detailed human movement behaviour in epidemiological models to incorporate spatial, social and demographic heterogeneity is demanding both in terms of necessary data and computational resources [2]. However, the first of these is likely to be addressed by the foreseeable growth in Big Data [32] and large databases that document human movement and behavioural patterns, such as data that is collated and managed by the retail [7], [8], [10] and communications sectors [5], [33]. Our ability to control and contain the spread of infectious disease will therefore continue to benefit from our growing capacity to predict human movement behavior and assess its impact on infection dynamics.

## Supporting Information

Text S1 Supporting Information Sections S1–S8.
(PDF)

Text S2 Supporting Information Section S9.
(PDF)
