# Supplementary material for: Strategies for Controlling Non-Transmissible Infection Outbreaks Using a Large Human Movement Data Set
Source: PLoS Comput Biol. 2014 Sep 11;10(9):e1003809. doi: 10.1371/journal.pcbi.1003809 (PMC4161289; doi:10.1371/journal.pcbi.1003809)
Supplement: Text S1 — Supporting Information Sections S1–S8. (PDF) [file pcbi.1003809.s001.pdf]

Supplementary Online Material (Sections S1-S8)

S1. Data describing spatial movement patterns of individuals in the population of Great Britain……………………………………………………………………………………………......2

S2. Approximating the likelihood of infection source locations………………………......7

S3. Movement probability as a function of distance from home……………………..........8

S4. MCMC results and convergence..10

S5. DIC values for models assuming different infection source locations..................14

S6. The distribution of the residential locations of the infected individuals……....15

S7. DIC maps for the outbreaks in Hereford and Barrow-in-Furness.........................16

S8. Maps of the predicted population at risk of infection..18

S6. References..19

S1. Data describing spatial movement patterns of individuals in the population of the Great Britain

This study obtained a database describing predicted travel patterns of the individuals comprising the populations of England, Scotland and Wales from the commercial consultancy, Oxford Retail Consultants (see section S8). The total area of England, Scotland and Wales is subdivided into hexagons of 500m diameter, giving a spatial resolution of approximately 21 million spatial units. The data includes estimates of the number of individuals of each demographic type who live in each hexagon, where the demographic type is defined by the age, gender and employment class to which the individual belongs. There are eight age classes (0-11, 11-15, 16-24, 25-34, 35-44, 45-54, 55-64 and >65 years of age) and five employment classes (full-time employed, part-time employed, unemployed, economically inactive or full-time education). In this study we consider only individuals older than 34 years of age because susceptibility to Legionnaires' disease is predominantly restricted to these older age groups [1-3]. Therefore the full-time student employment class was not used in this study because very few individuals older than 34 are in this category.

For each demographic type and hexagon of residence, the database provides estimates of the probability that the individuals within this category visit locations in the landscape for activities that are categorized as work, education at schools or universities, shopping for food and non-food consumables and other unknown activities (see Figures S1.1 and S1.2). The probabilities depend on the time of the week in which the activity is undertaken. The week is divided into 28 components, four components for each day, which are defined as night (8pm-6am), peak morning (6am – 10am), day (10am-4pm) and peak evening (4pm-8pm). An example of how the probability of engaging in different activities varies throughout the week is given in Figure S1.3.

The probability that individual *i* is present in hexagon *x* during day part *d* given their demographic type ϕ_i and hexagon of residence H_i, $P^d(x|H_i,\phi_i)$, can be estimated by the sum:

$$P^d(x|H_i,\phi_i)=\sum_{\forall k}P(x|H_i,\phi_i,k)P^d(k|\phi_i) \qquad \text{(S1.1)}$$

which we decompose into the probability of undertaking a particular activity *k* at a given time, $P^d(k|\phi_i)$, and the movement probability given the activity $P(x|H_i,\phi_i,k)$. An estimate of the probability that an individual is at home on day part *d* is included in the set of probabilities $P^d(k|\phi_i)$.

Spatial patterns of visitation vary considerably depending on the type of activity for which individuals travel. The weekly averages of the expected number of individuals who reside in a given hexagon that visit each destination are plotted in Figure S1.1 for a subset of the data that includes individuals who travel to (or reside in) locations within the city of Stoke-on-Trent, England. Shopping destinations are visited by a relatively large number of individuals, which reflects the smaller number of potential shopping destinations in the landscape compared to the number of work destinations (see the examples in Figure S1.2). Preferred shopping destinations are generally closer to home than work destinations (see Figures S1.1, S1.2 and Section S3). The range of work destinations is generally smaller for older demographic types as fewer of these individuals are employed (eg. Figure S1.2D).

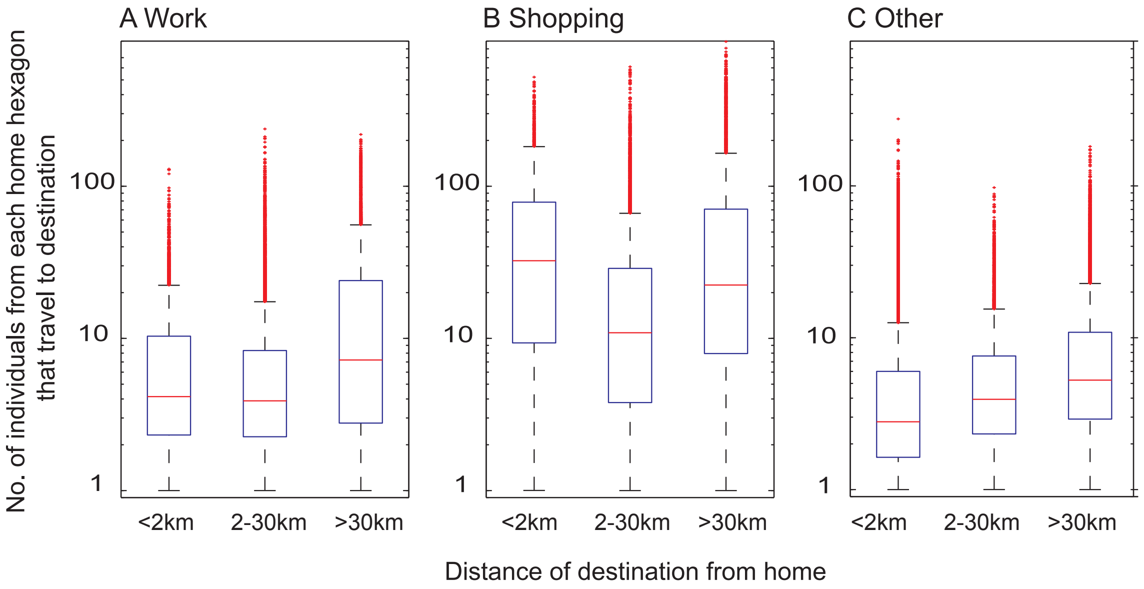

Figure S1.1. The box-whisker distributions of the expected number of individuals residing in each hexagon who travel to other hexagons at different distances from home. A subset of the data including 1027622 individuals older than 34 years of age whose travel destinations are within Stoke-on-Trent is shown. A. Travel is for work activity B. Travel is for shopping activity C. Travel is for unknown activity and the destinations are determined by extrapolation of the source data (see section S1.1).

S1.1 Data extrapolation to estimate additional movement behaviours

The locations represented in the Great Britain human movement database estimate destinations to which individuals travel for work, shopping or education related activities but do not describe the complete travel activity of individuals. The probabilities $P^d(x|H_i,\phi_i)$ (eqn S1.1) summed across all destinations *x* for each individual average about 0.8 across all individuals. To account for missed movements the probabilities $P^d(x|H_i,\phi_i)$ were extrapolated to include the chance of visiting additional local destinations. The extrapolation procedure that assumes that each individual is likely to visit areas nearby to the destinations included in the database for the individual. This assumption allows the representation of a greater degree of overlap in the destinations visited by multiple individuals than that represented in the database, which can be important to identifying sources of infection common to multiple individuals.

Each individual was assigned probabilities of visiting an additional set of destinations defined as those within a given radius *r* of the destinations included in the database for that individual. For the work and shopping destinations included in the database *r* was set to 500m, or 1 hexagon. For home locations a larger radius of *r*=2500m (or 5 hexagons) was chosen because our data indicates that individuals spent much more time at home that at any other given location (e.g. see Figure S1.3).

Denote the hexagon centroids of the destinations represented in the human movement database as x_c and the hexagon centroids that are located within a distance *r* from x_c as as y_c, where the distance must be greater than zero to exclude the central hexagon from the set of locations y_c. For each individual of demographic type ϕ_i residing in hexagon H_i, a probability of visiting each additional destination y_c on a day part that is dependent on the probability of visiting the central location x_c, was assigned. This

probability, denoted $R^d(y_c|H_i,\phi_i,x_c)$, is proportional to the weekly average probability that the individual is present in location x_c, denoted $\bar{P}(x_c|H_i,\phi_i)$, the inverse squared distance of y_c from x_c, d_c^{-2}, and the expected average number of individuals present at the destination y_c, z_{y_c}, where

$$z_{y_c} = \sum_i \bar{P}(y_c|H_i,\phi_i) \quad \text{(S1.2)}$$

The extrapolated probabilities were normalized so that the weekly average of the probabilities that an individual is present in a destination summed across all destinations *x* was equal to 1 for each individual, including the destinations added by the extrapolation. Thus the probability that the individual visits a location y_c on a day part during localized movements in the vicinity of the destinations included in the human movement database is

$$R^d(y_c|H_i,\phi_i) = \sum_{x_c} R^d(y_c|H_i,\phi_i,x_c) \quad \text{(S1.3)}$$

where

$$R^d(y_c|H_i,\phi_i,x_c) = \frac{\bar{P}(x_c|H_i,\phi_i)}{\sum_{x_c}\bar{P}(x_c|H_i,\phi_i)}\left(1-\sum_{x_c}\bar{P}(x_c|H_i,\phi_i)\right)\frac{w_{y_c}}{\sum_{y_c} w_{y_c}} \quad \text{(S1.4)}$$

where $w_{y_c} = z_{y_c} d_c^{-2}$. This calculation gives the same value of $R^d(y_c|H_i,\phi_i)$ for all day parts (because it is based on weekly average probabilities), which does not affect the calculation of the likelihood l_s (eqn 2) that depends only on the sum of the movement probabilities over all day parts.

The extrapolation procedure results in several extra destinations being added to an individual's spatial movement profile (e.g. see Figure S1.2C). The rates of visitation received by a destination due to these unknown activities is relatively low (e.g. see Figure S1.1C).

S1.2 Estimating individual movement probabilities from the Great Britain human movement database

The probability that individual *i* is present in a location *S* on day part *d* is separated into the probabilities for activities within and outside the home, $P_{i,h}^d(S)$ and $P_{i,v}^d(S)$.

We assume that the probabilities for engaging in activities in location *S* outside the home, $P_{i,v}^d(S)$, are made up of three quantities,

$$P_{i,v}^d(S) = P_v^d(S|H_i,\phi_i) + R^d(S|H_i,\phi_i) + \varepsilon z_s \quad \text{(S1.5)}$$

The first term, $P_v^d(S|H_i,\phi_i)$, is the estimate given in the human movement database of the probability that individual *i* was present location *S* on day part *d* for any activity that does not take place at home. In the second term $R^d(S|H_i,\phi_i)$ is the estimated probability that individual *i* visited location *S* on day part *d* during localized movements in the vicinity of the destinations present in the database. Values of $R^d(S|H_i,\phi_i)$ were estimated by the extrapolation procedure described above (section S1.1). The third term accounts for unknown movements of the individuals which are modelled by assuming that each individual has a probability of visiting any hexagon that is proportional to the average expected number of individuals that are present in the hexagon, z_s (see eqn S1.2). This term ensures that all individuals have a non-zero probability of occupying any location in

the landscape that is not vacant. The value of constant of proportionality, ε, is unknown, and was set to a small number (10^{-16}) so that values of the third term in eqn (1.5) are no larger than about 3 orders of magnitude smaller than the first and second terms. The probability that an individual is at home in location *S* on day part *d*, is $P_{i,h}^{d}(S)$ simply estimated by $P_{h}^{d}(S|H_{i},\phi_{i})$, the estimate given in the human movement database.

S1.3 Estimating individual movement probabilities from reported travel histories
For data richness Level 1 considered in this analysis, travel history data was used to estimate the movement probabilities $P_{i,h}^{d}(S)$ and $P_{i,v}^{d}(S)$ for infected individuals. The travel histories for each individual do not account for their locations at all times, therefore travel history data was extrapolated as described above (section S1.1) to estimate the larger set of destinations that the individuals may have visited. Travel history data for each individual is only available for a 1-4 week portion of the total censoring time T_c. We therefore assume that the individuals repeated the same movement patterns with a weekly period throughout the censoring time.

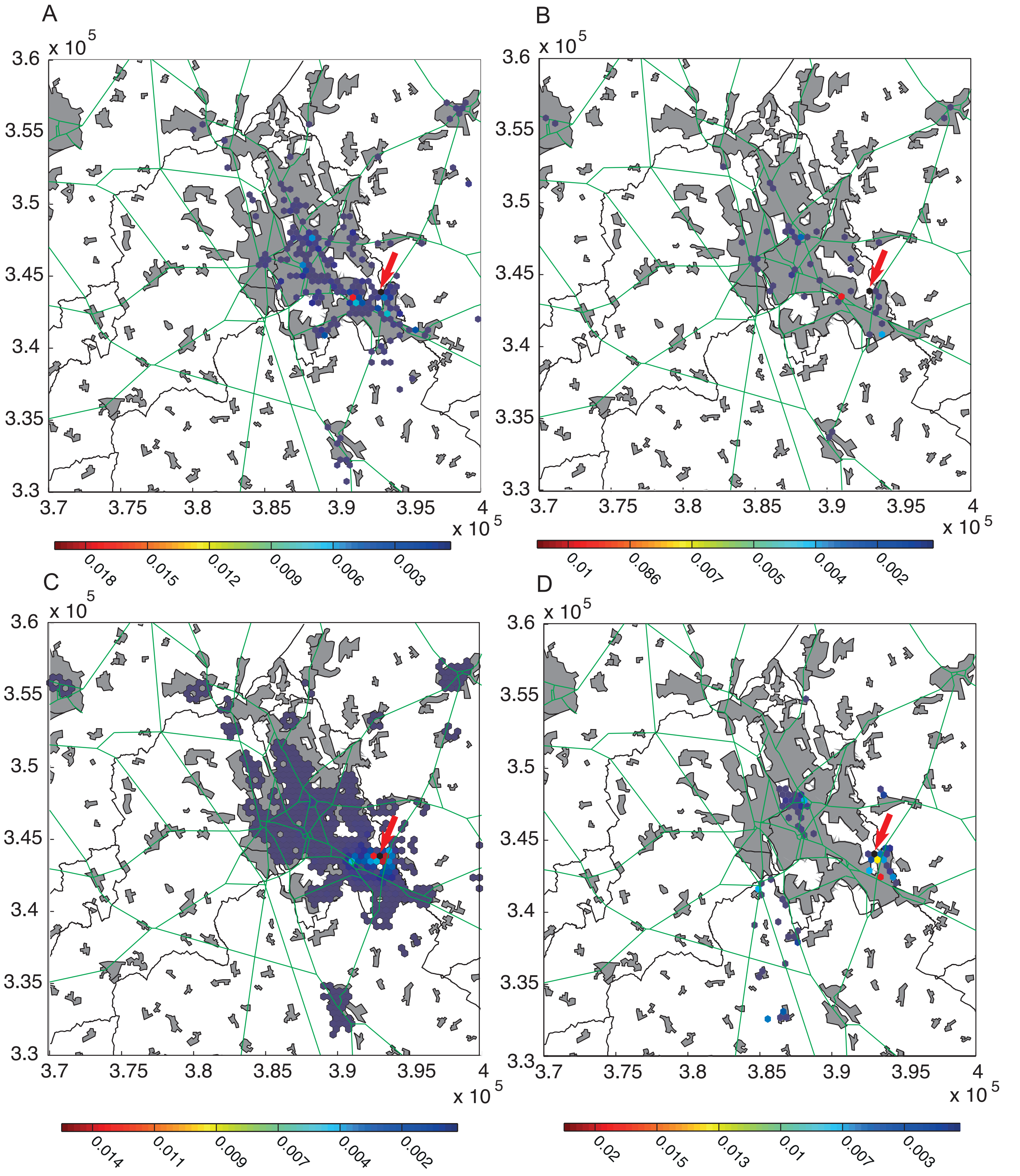

Figure S1.2. Hexagons containing the predicted destinations visited by an individual residing within the black hexagon (indicated by the red arrow) located in Stoke-on-Trent, England. Hexagons are coloured according to the weekly average probability of occupying the hexagon in a day part. In A, B and C the individual's demographic type is a full-time employed female aged between 35-44 years old and in D the demographic type is a part-time employed male aged 55-64 years old. Destinations for different activities are shown: Work destinations (A, D), shopping destinations (B) and destinations for unknown activities estimated using the extrapolation procedure described in section S1.1 (C).

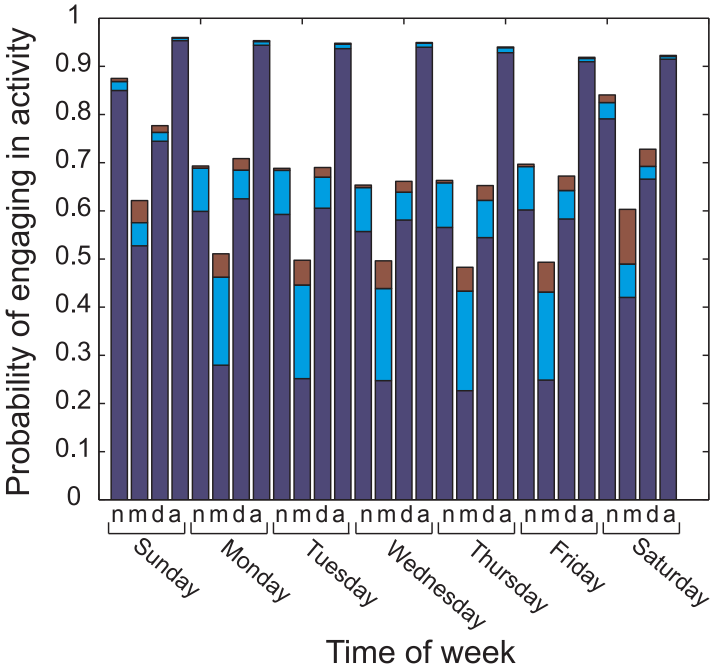

Figure S1.3. The probability of engaging in an activity at different times of week time of the week. Activities shown include home activities (dark blue portion), work (light blue portion) and shopping (brown portion). Probabilities for the day parts of each day are shown (n=night, m=peak morning, d=day and a=peak evening). Data correspond to a female aged 35-44 in full time employment.

S2. Approximating the likelihood of infection source locations

Assume that a single location *S* is the source of infection with a non-transmissible pathogen and that the infection rate within the source hexagon is a constant λ_s per week. Individuals who reside in hexagon *S* are allowed to experience a different infection risk when they are at home, given by $\beta_s\lambda_s$ where β_s is a constant. The probability that individual *i* becomes infected on day part *d*, p_i^d, is

$$p_i^d = P_{i,v}^d(S)(1-e^{-\lambda_s\tau}) + P_{i,h}^d(S)(1-e^{-\beta_s\lambda_s\tau}) \qquad \text{(S2.1)}$$

where τ is the duration of a day part. The probability of becoming infected during the exposure period is then

$$p_i = 1-\prod_{d\in D}(1-P_{i,v}^d(S)(1-e^{-\lambda_s\tau}) - P_{i,h}^d(S)(1-e^{-\beta_s\lambda_s\tau})) \qquad \text{(S2.2)}$$

where *D* is the set of day parts in the period of exposure. Now assuming $\lambda_s\tau$ is small we approximate $1-e^{-\lambda_s\tau}$ and $1-e^{-\beta_s\lambda_s\tau}$ by $\lambda_s\tau$ and $\beta_s\lambda_s\tau$ respectively, and $P_{i,v}^d(S)\lambda_s\tau + P_{i,h}^d(S)\beta_s\lambda_s\tau$ by $1-e^{-P_{i,v}^d(S)\lambda_s\tau-\beta_s P_{i,h}^d(S)\lambda_s\tau}$, which gives

$$p_i = 1-\exp\left(-\lambda_s\tau\sum_{d\in D}(P_{i,v}^d(S)+\beta_s P_{i,h}^d(S))\right) \qquad \text{(S2.3)}$$

Now assume that our infection data describe a set of infected individuals *I* and the remaining set of uninfected individuals *U* in the population over a censoring time T_c. The likelihood l_s of the data is

$$l_s = \prod_{i \in I} p_i \prod_{j \in U} (1 - p_j), \qquad D = T_c$$ (S2.4)

Using (S2.3), the log likelihood, L_s, is

$$L_s = \sum_{i \in I} \log(\lambda_s \tau P_{i,v}(S) + \beta_s \lambda_s \tau P_{i,h}(S)) + \sum_{j \in U} -\lambda_s P_{j,v}(S)\tau - \beta_s \lambda_s P_{j,h}(S)$$ (S2.5)

letting $P_{i,v}(S) = \sum_{d \in D} P^d_{i,v}(S)$ and $P_{i,h}(S) = \sum_{d \in D} P^d_{i,h}(S)$ and approximating $1 - e^{-P_{i,v}(S)\lambda_s\tau - P_{i,h}(S)\beta_s\lambda_s\tau}$ by $P_{i,v}(S)\lambda_s\tau + \beta_s P_{i,h}(S)\lambda_s\tau$. The value of λ_s that maximizes the log likelihood is given by partial differentiation with respect to λ_s to give

$$\sum_{i \in I} \frac{1}{\lambda_s} = \sum_{j \in U} P_{j,v}(S)\tau + P_{j,h}(S)\beta_s\tau$$ (S2.6)

so that the maximum likelihood value λ^* is

$$\lambda^*_s = n_I / \sum_{j \in U} P_{j,v}(S)\tau + \beta_s P_{j,h}(S)\tau$$ (S2.7)

where n_I is the number of cases. Substituting this expression for λ^* into the equation for L_s (eqn S2.5) gives

$$L^*_s = n_I(\log n_I - 1) + \sum_{i \in I} \log \frac{P_{i,v}(S) + \beta_s P_{i,h}(S)}{\sum_{j \in U} P_{i,v}(S) + \beta_s P_{i,h}(S)}$$ (S2.8)

which shows that the maximum likelihood value depends on the expected amount of time that each infected individual spends in location *S* relative to the total expected amount of time spent in location *S* by all uninfected individuals. The same methodology can applied to the case where there are multiple locations containing sources of infection to estimate the maximum likelihood values of the infection rates for each source.

In this study we used a Bayesian model to analyse the log likelihood function (eqn S2.5). Given that this model assumed uniform prior distributions $U(0,1)$ for the parameters λ_s and $\beta_s\lambda_s$, the maximum likelihood estimate λ^*_s is the maximum a posterior probability (MAP) estimate of the mode of the posterior distribution of λ_s [4]. We make use of the analytic expression for λ^*_s to chose the value of the unknown movement probabilities for uninfected individuals, $P^d_{i,v}(S)$, for the lowest level of movement data richness considered in this analysis (Level 3). These probabilities are chosen so to give equal estimates of the posterior modes of λ_s and $\beta_s\lambda_s$ for data richness Level 3 and the higher data richness Level 2 (see section S3).

Further, we use the estimate of λ^*_s to verify that the MCMC Gibbs sampling algorithm converges to the analytic solution (see section S4).

S3. Movement probability as a function of distance from home

For data richness Level 3 described in the main text, we used the power law kernel of the form $f(d) \sim 1/(1 + (d/a)^b)$ to predict the weekly average probability that infected individuals occupy a location a distance *d* from home during a day part, *f(d).* Thus, the probability that an individual *i* visits location *S* on a day part *d,* $P^d_{i,v}(S)$, is estimated by

$$P^d_{i,v}(S) = f(d_s)$$ (S3.1)

where d_s is the distance between the hexagon in which the individual resides, H_i, and the infection source S. A kernel of the same form was used by Ferguson et al. [5] to model workplace commuting journeys and was found to provide an accurate fit to empirical data. In this study the model was fit to data from our Great Britain human movement database. The kernel was fit separately to subsets of the data corresponding to journeys for work and non-work related purposes and different regions of the country. The regions corresponded to the locations of the three outbreaks of Legionnaire's disease analysed in this study. Regions containing hexagons that could potentially contain an infection source were defined by bounding boxes around each city in which an outbreak occurred. Individuals that either resided in or visited the hexagons inside the bounding boxes were included in the data subsets.

The power law functions obtained by non-linear least squares fitting to the data subsets fit the data with high precision (Figure S3.1). Non-work journeys are distributed closer to home than work journeys (Figure S3.1).

The probabilities of uninfected individuals visiting any location in the landscape is unknown, and was set to a constant value. We chose this value to give agreement between the infection rates λ_s and $\beta_s\lambda_s$ for this data richness level and a higher richness level (Level 2) for the hexagon containing the true infection source. Agreement was measured by comparing the modes of the posterior distributions of λ_s and $\beta_s\lambda_s$ obtained for data richness levels 2 and 3 (see section S2).

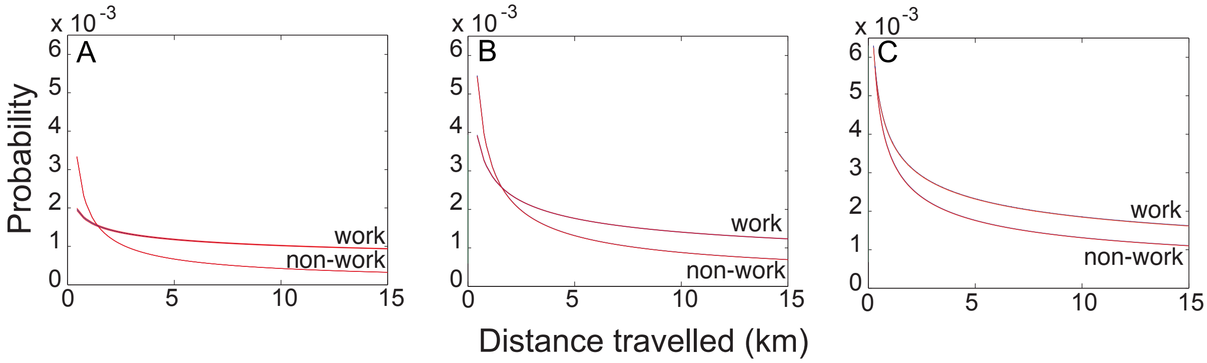

Figure S3.1. The weekly average probability of occupying a destination during a day part as a function of distance from home, estimated by fitting power law functions to data describing the movement patterns of individuals in Great Britain. Functions were fitted separately to subsets of the data: A. 1027622 individuals whose destinations are within Stoke-on-Trent (for work destinations a = 3.5 x 10^{-7} km, b = 0.4, for non-work destinations a = 7.5 x 10^{-5} km, b = 0.66) B. 768606 individuals whose destinations are within Hereford (for work destinations a = 1.9 x 10^{-8} km, b = 0.33, for non-work destinations a = 5.7 x 10^{-5} km, b = 0.58) C. 551765 individuals whose destinations are within Barrow-in-Furness (for work destinations a = 4.6 x 10^{-8} km, b = 0.33, for non-work destinations a = 1.7 x 10^{-6} km, b = 0.43). Blue lines show the fitted function and red lines show 95% confidence intervals.

S4. MCMC results and convergence diagnostics

Posterior distributions

For the three outbreaks (in Stoke-on-Trent, Hereford and Barrow-in-Furness) the posterior distributions of the infection rates λ_s and $\beta_s\lambda_s$ are approximately normally distributed and centered close to the analytic approximation to the mode (given by eqn S2.7). For example, Figure S4.1 shows these posterior distributions for hexagon containing the true source. For the Stoke-on-Trent outbreak the infection rate $\beta_s\lambda_s$ was dropped from the model because the source hexagon had no residents.

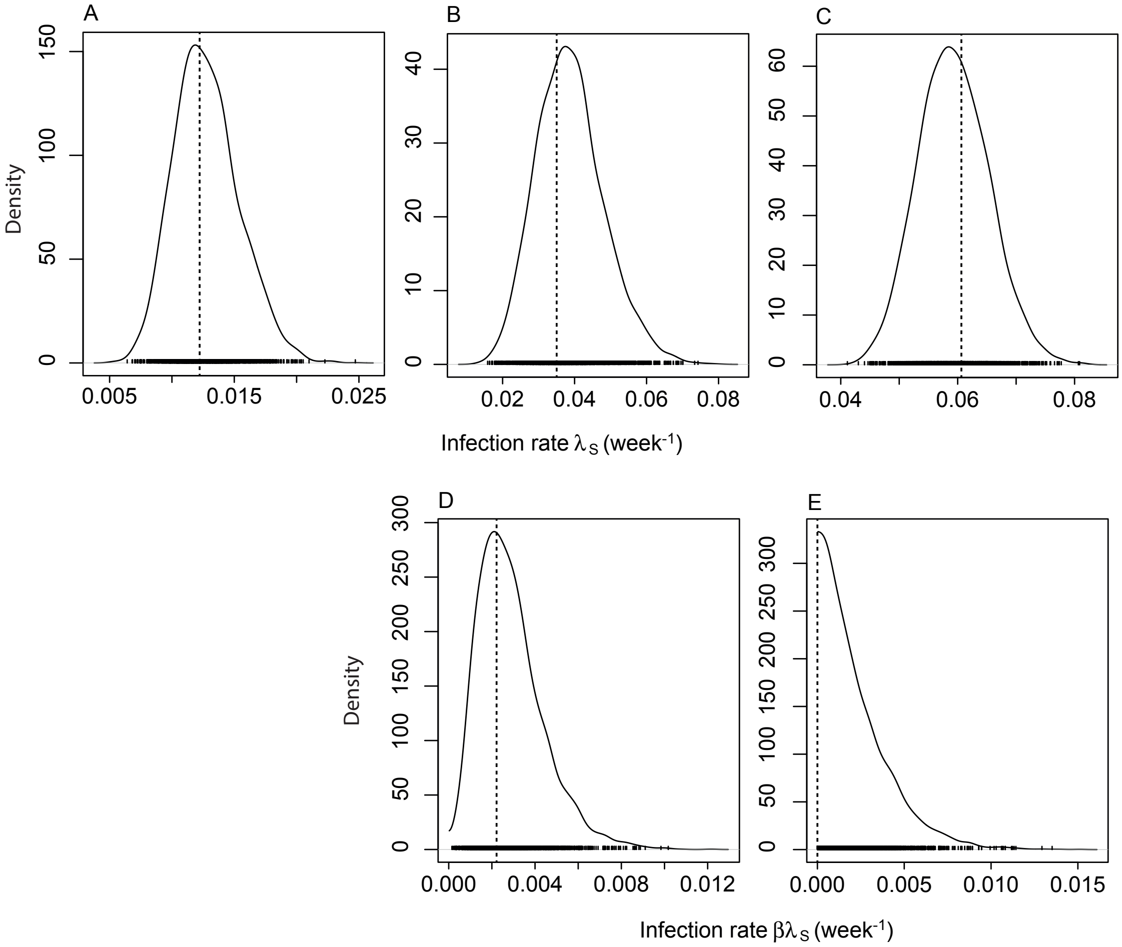

Figure S4.1. Posterior distributions of λ_s and $\beta_s\lambda_s$ for the hexagon containing the true source of infection for the outbreaks in Stoke-on-Trent (A), Hereford (B,D) and Barrow-in-Furness (C,E). The dotted lines show the value of the analytic approximation given in eqn S2.7. Movement probabilities for infected and uninfected individuals were estimated assuming richness Level 2 of the data describing human movement patterns i.e. the Great Britain human movement database was used to estimate all movements.

Convergence diagnostics

To test whether the Gibbs sampling algorithm converged to a unique posterior distribution we used three measures of assessment. Firstly, agreement between the modes of the posterior distributions of the infection rates λ_s and $\beta_s\lambda_s$ and the analytic approximation given in eqn S2.7 was verified (Figure S4.1). Secondly, the trace plots of λ_s and $\beta_s\lambda_s$ were visually examined to verify that the samples were well mixed. The

Gibbs sampler was run using 3 chains, with 3000 iterations per chain and discarding the first 1000 iterations. The initial values for each chain were set by sampling randomly from the prior distributions for each parameter (uniform priors $U(0,1)$ in both cases). Examples of trace plots are given in Figures S4.2, S4.3 and S4.4. Thirdly, Gelman-Rubin plots were visually examined to verify that the Gelman-Rubin shrink factor converged to 1 (Figures S4.2, S4.3 and S4.4).

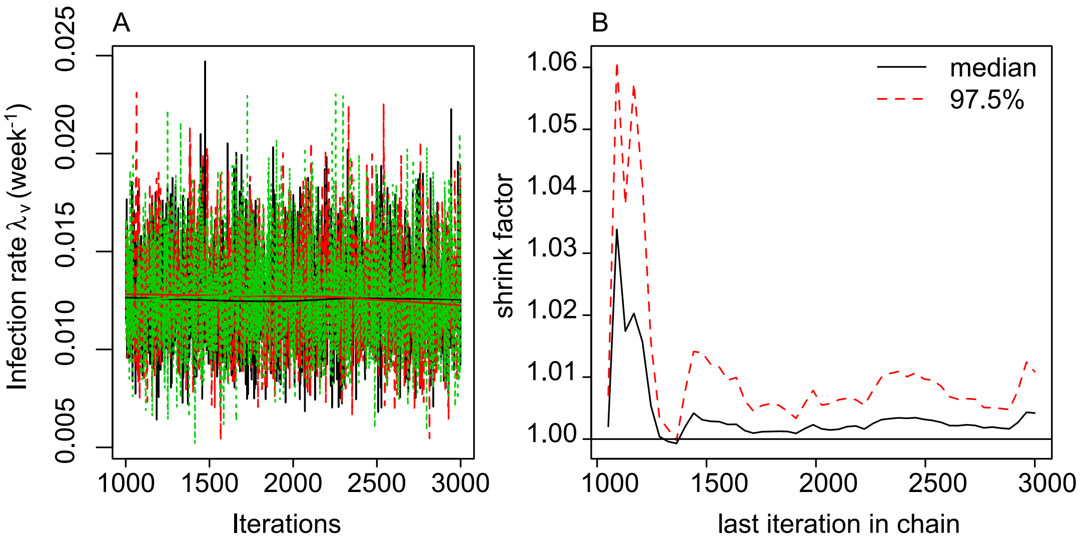

Figure S4.2. Trace plot (A) and the Gelman-Rubin plot (B) for the infection rate λ_s for the Stoke-on-Trent outbreak. The model assumes that the infection source is in the hexagon containing the true source. Movement probabilities for infected and uninfected individuals were estimated assuming richness Level 2 of the data describing human movement patterns i.e. the Great Britain human movement database was used to estimate all movements.

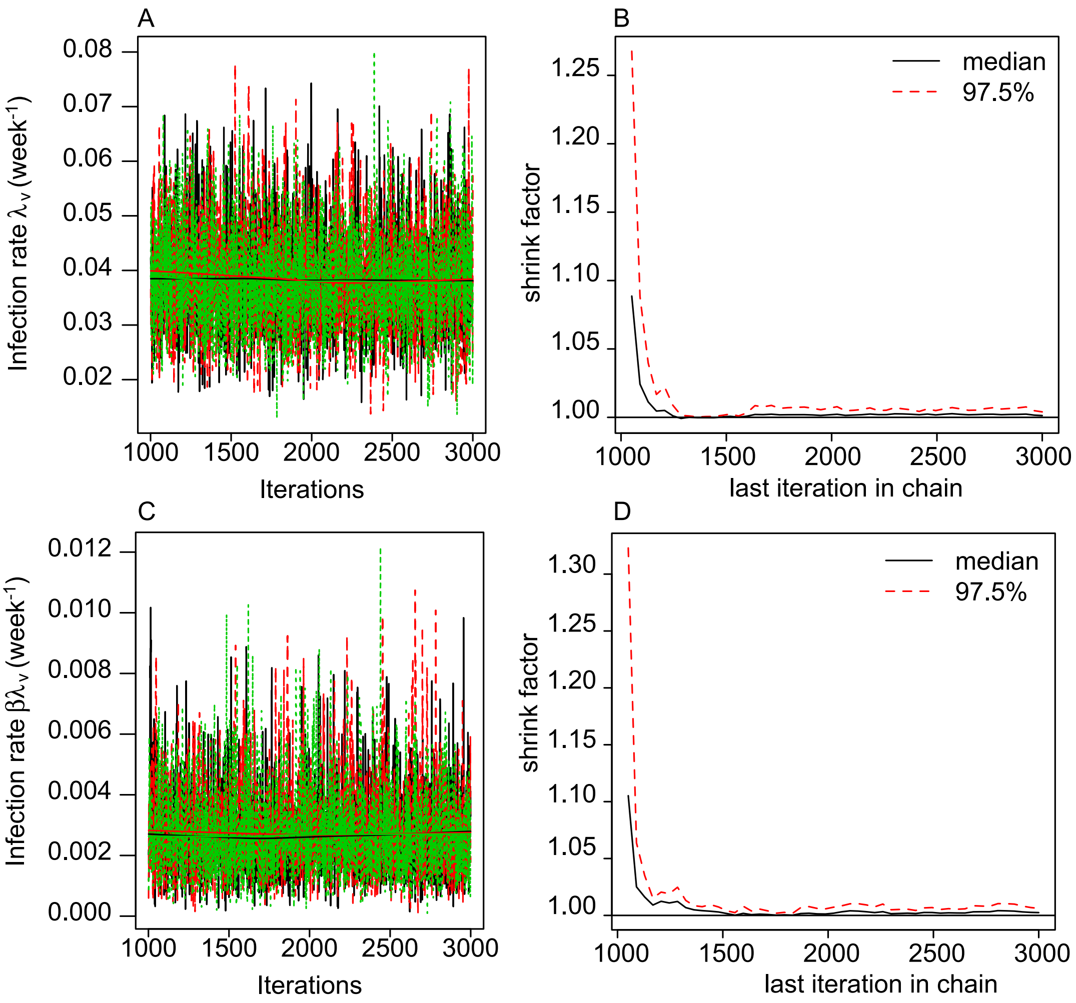

Figure S4.3. Trace plots (A,C) and the Gelman-Rubin plots (B,D) for the Hereford outbreak. Panels A-B show λ_s and panels C-D show $\beta_s\lambda_s$. The model assumes that the infection source is in the hexagon containing the true source. Movement probabilities for infected and uninfected individuals were estimated assuming richness Level 2 of the data describing human movement patterns i.e. the Great Britain human movement database was used to estimate all movements.

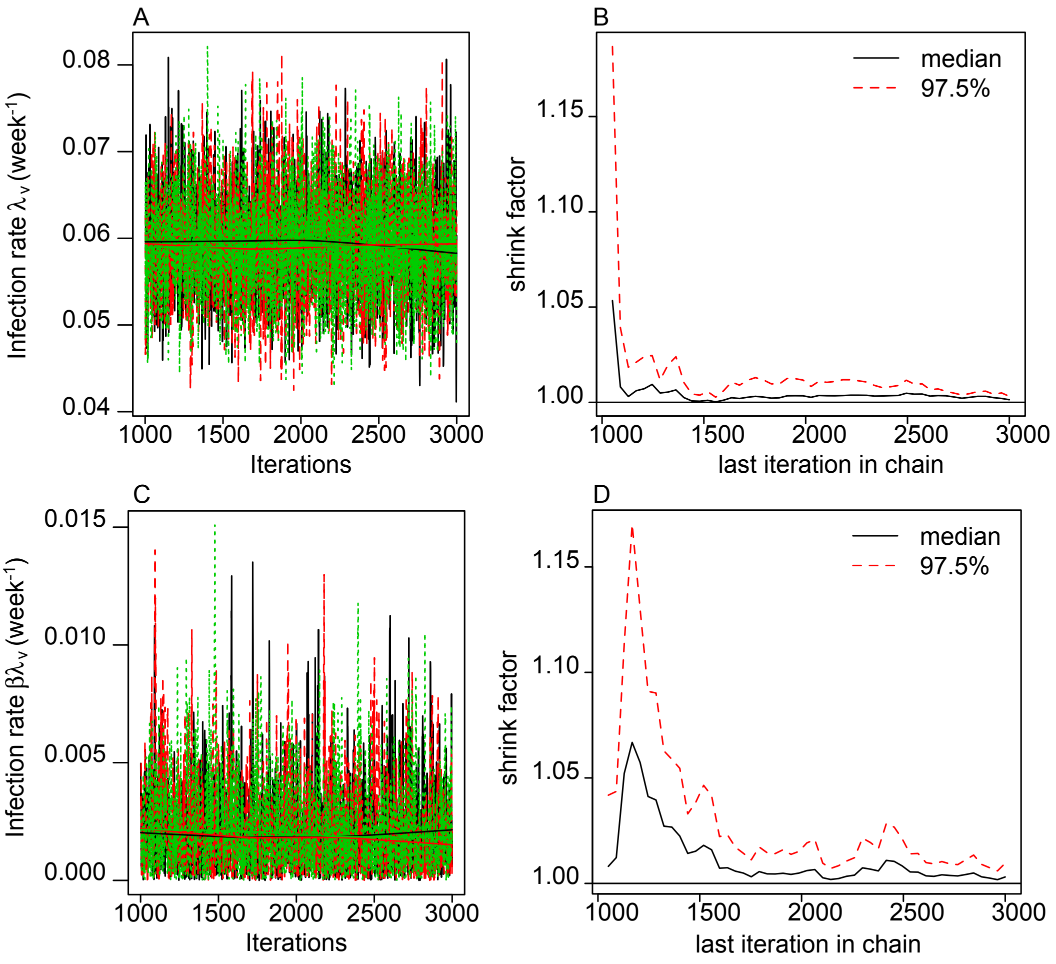

Figure S4.4. Trace plots (A,C) and the Gelman-Rubin plots (B,D) for the Barrow-in-Furness outbreak. Panels A-B show λ_s and panels C-D show $\beta_s\lambda_s$. The model assumes that the infection source is in the hexagon containing the true source. Movement probabilities for infected and uninfected individuals were estimated assuming richness Level 2 of the data describing human movement patterns i.e. the Great Britain human movement database was used to estimate all movements.

S5. DIC values for models assuming different infection source locations

Table S5.1. The DIC values and the distance from the true infection source for the top 4 ranking models. Each model corresponds to a different assumed infection source location for a given outbreak and human movement data richness level.

Outbreak	Data richness level	Rank of model	DIC	Distance from true source (km)
Stoke-on-Trent	1	1	531.8	0
Stoke-on-Trent	1	2	559.4	1.1
Stoke-on-Trent	1	3	631.7	0.4
Stoke-on-Trent	1	4	693.7	0.8
Stoke-on-Trent	2	1	493.8	1.3
Stoke-on-Trent	2	2	500.5	1.9
Stoke-on-Trent	2	3	507.5	1.7
Stoke-on-Trent	2	4	518.0	3.9
Stoke-on-Trent	3	1	624.4	1.9
Stoke-on-Trent	3	2	630.2	4.2
Stoke-on-Trent	3	3	637.1	1.7
Stoke-on-Trent	3	4	639.6	1.6
Hereford	2	1	328.0	1.3
Hereford	2	2	329.3	0
Hereford	2	3	329.9	1.6
Hereford	2	4	331.2	0.4
Hereford	3	1	454.8	0
Hereford	3	2	457.7	1.9
Hereford	3	3	466.6	1.5
Hereford	3	4	470.6	2.4
Barrow-in-Furness	2	1	1400.1	0.4
Barrow-in-Furness	2	2	1447.5	0.4
Barrow-in-Furness	2	3	1457.5	0.4
Barrow-in-Furness	2	4	1460.3	0.8
Barrow-in-Furness	3	1	2276.2	0.9
Barrow-in-Furness	3	2	2292.5	0.4
Barrow-in-Furness	3	3	2293.7	1.3
Barrow-in-Furness	3	4	2303.4	1.1

S6. The distribution of the residential locations of the infected individuals

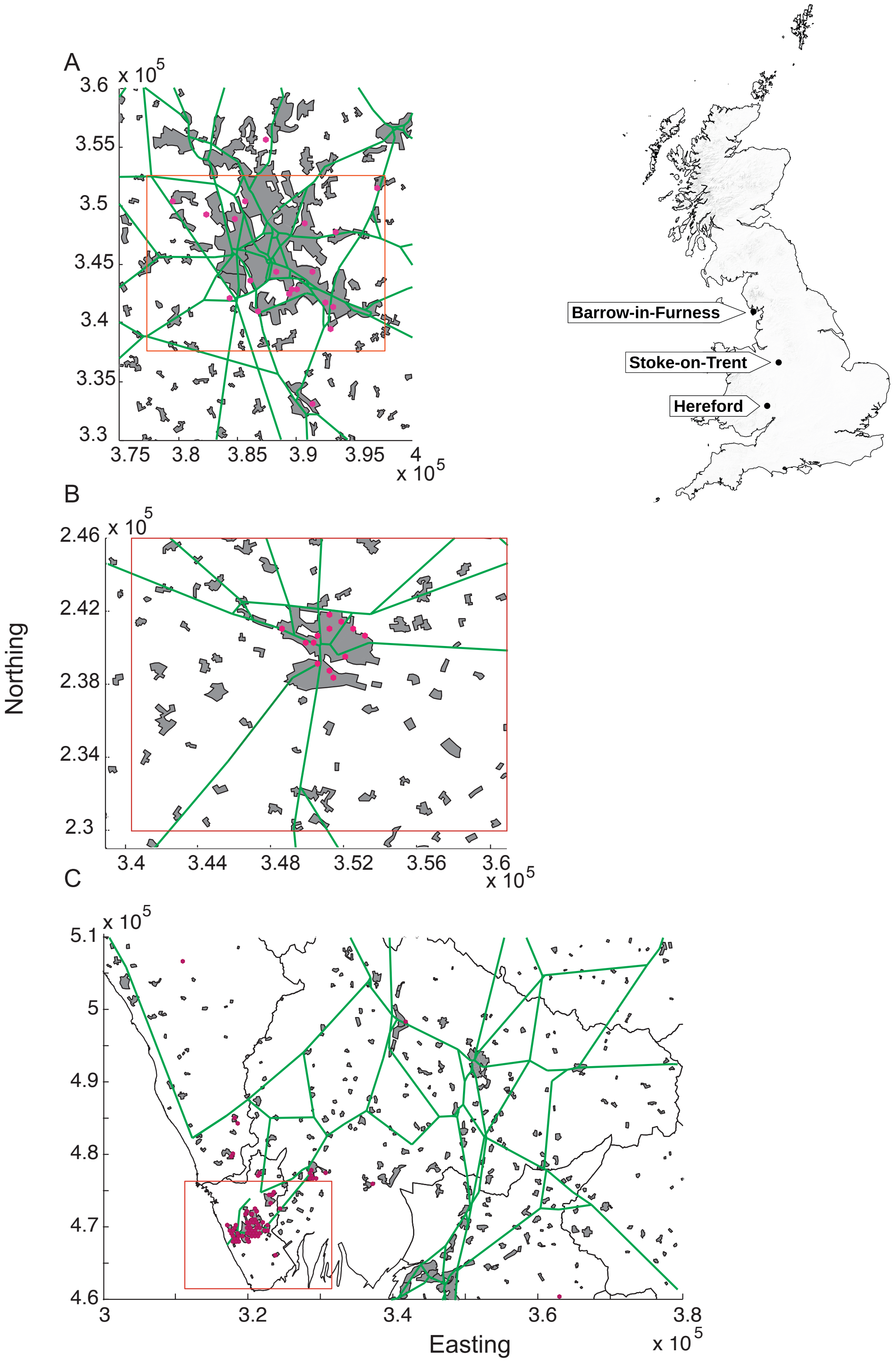

Figure S6.1. The distribution of the hexagons containing the residential locations of the infected individuals associated with the outbreaks of Legionnaires' disease in A. Stoke-on-Trent B. Hereford and C. Barrow-in-Furness. The red boxes show the 20 x 15 km areas that are depicted in Figure 2 in the main text and in Figures S6.1 and S6.2. The gray shaded areas indicate urban areas, green lines indicate major roads and the hexagons are coloured pink. Contains Ordinance Survey Data © Crown Copyright and database right 2014.

S7. DIC maps for the outbreaks in Hereford and Barrow-in-Furness

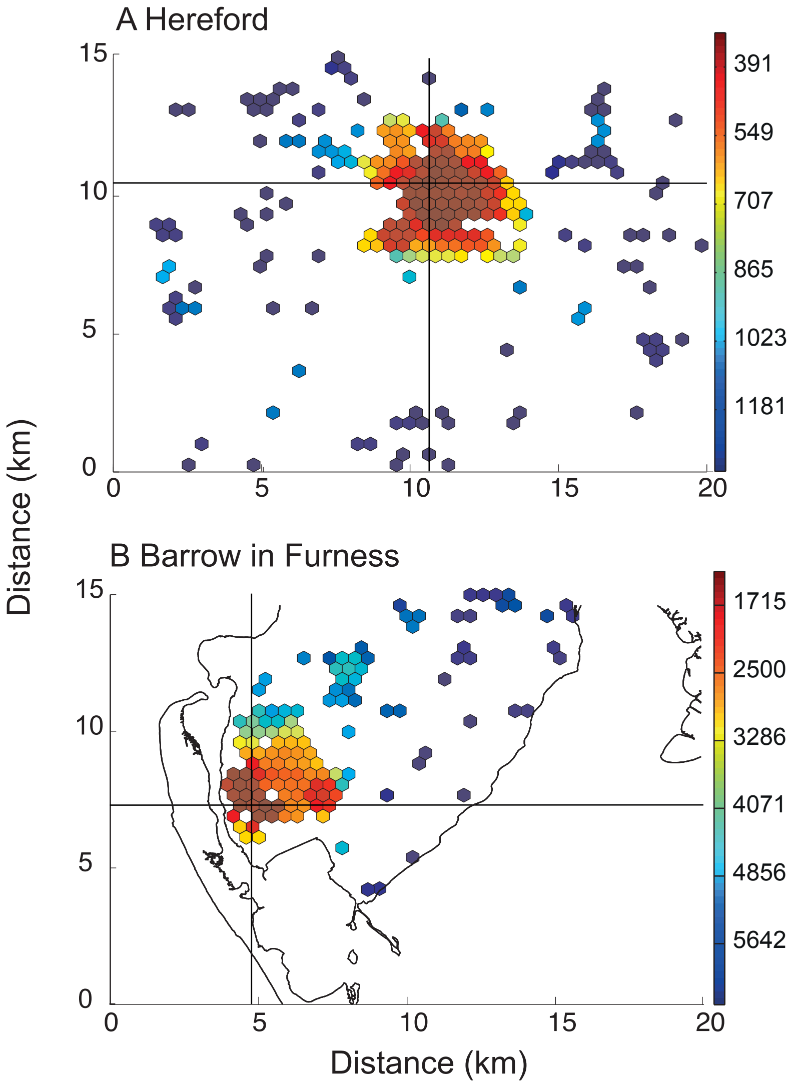

Figure S7.1 The DIC values for models assuming different locations of the infection source for the outbreaks in A. Hereford and B. Barrow-in-Furness. Level 2 of the richness of data describing human movements is shown. The black lines intersect at the location of the true infection source. Town features are not shown to preserve anonymity. In B, the black border shows the coastline.

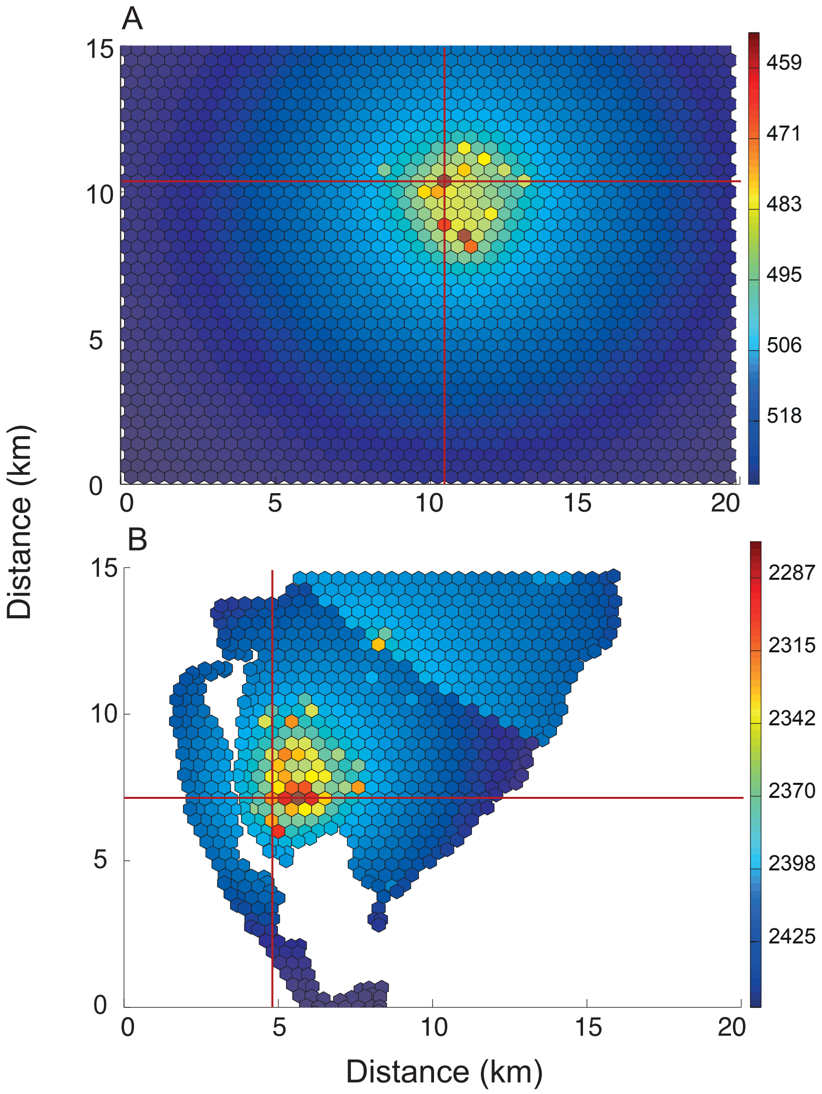

Figure S7.2 The DIC values for models assuming different locations of the infection source for the outbreaks in A. Hereford and B. Barrow-in-Furness. Level 3 of the richness of data describing human movements is shown. The black lines intersect at the location of the true infection source. Town features are not shown to preserve anonymity. In B, the black border shows the coastline.

S8. Maps of predicted population at risk of infection

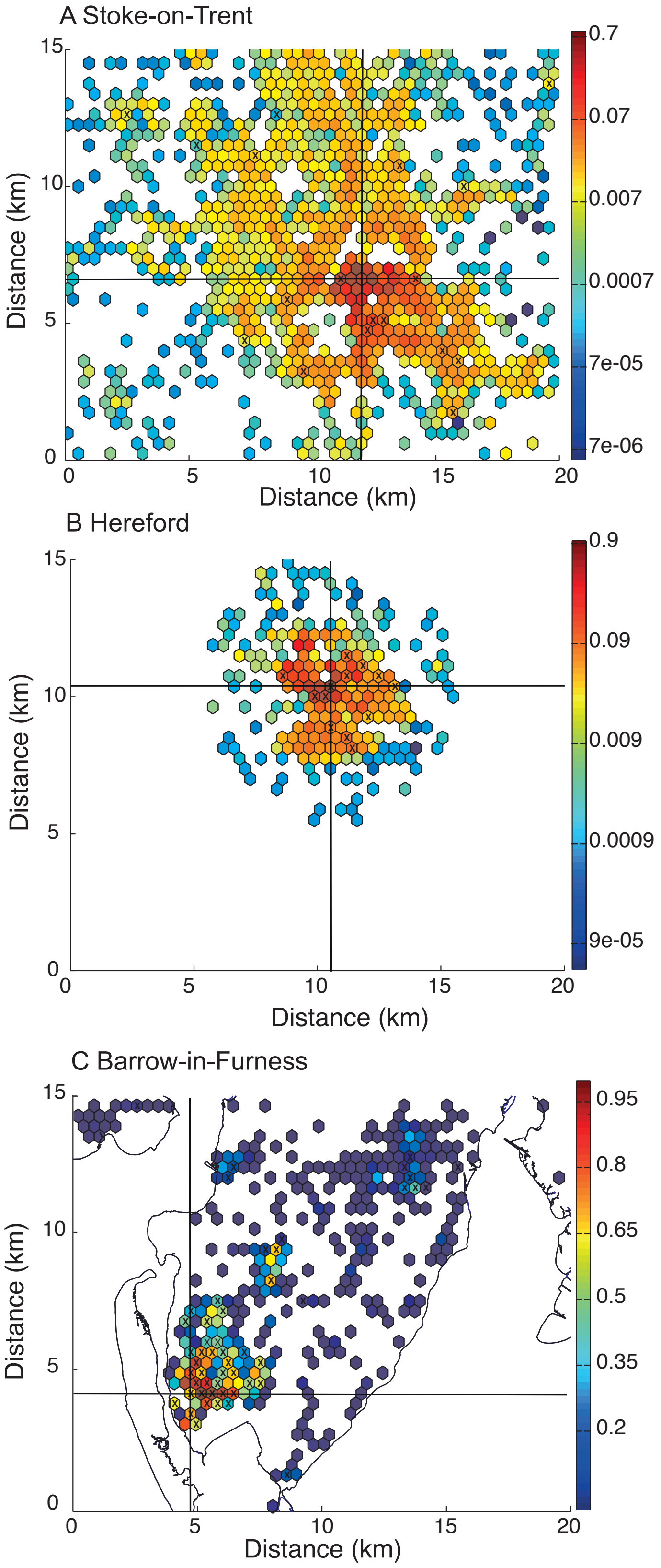

Figure S8.1 The predicted probabilities that a hexagon contains the home location of at least one case, $p_{+.H}$, for A. Stoke-in-Trent, B. Hereford and C. Barrow-in-Furness. Crosses show the hexagons containing observed case home locations. The black lines intersect at the location of the true infection source. In C, the black boundary shows the coastline.

References

1. Benin AL, Benson RF, Besser RE (2002) Trends in legionnaires disease, 1980-1998: Declining mortality and new patterns of diagnosis. Clin Infect Dis 35: 1039-1046.
2. Fields BS, Benson RF, Besser RE (2002) Legionella and Legionnaires' disease: 25 years of investigation. Clin Microbiol Rev 15: 506-526.
3. Garcia-Fulgueiras A, Navarro C, Fenoll D, Garcia J, Gonzalez-Diego P, et al. (2003) Legionnaires' disease outbreak in Murcia, Spain. Emerg Infect Dis 9: 915-921.
4. Berger JO (1985) Statistical decision theory and Bayesian Analysis New York: Springer-Verlag.
5. Ferguson NM, Cummings DAT, Fraser C, Cajka JC, Cooley PC, et al. (2006) Strategies for mitigating an influenza pandemic. Nature 442: 448-452.
