# Supplementary material for: Strategies for Controlling Non-Transmissible Infection Outbreaks Using a Large Human Movement Data Set
Source: PLoS Comput Biol. 2014 Sep 11;10(9):e1003809. doi: 10.1371/journal.pcbi.1003809 (PMC4161289; doi:10.1371/journal.pcbi.1003809)
Supplement: Text S2 — Supporting Information Section S9. (PDF) [file pcbi.1003809.s002.pdf]

Supplementary Online Material (Section S9)

S9. The Oxford Retail Consultants' Great Britain human movement database......2

References..5

S9. The Oxford Retail Consultants' Great Britain human movement database

In this study we utilize a human movement database, termed the Great Britain human movement database, that has been developed for this project by Oxford Retail Consultants. A range of similar databases are in operation or being created (see [1,2]), with one being developed for use by UK government agencies [3]. The continued growth in these databases is facilitated by computational advances in the storage and processing of data. Therefore while the Great Britain human movement database is key to the working of our model, we believe that any similarly detailed data would provide similar benefits.

While the precise formulation, parameters and underlying data used to derive the human movement database is commercially sensitive, the broad approach is standard [1,2]. The area of England, Wales and Scotland is overlaid by a hexagonal lattice, with each hexagonal cell having a diameter of 500 metres. It is this hexagonal lattice of approximately 21 million cells that forms the basis of all the underlying spatial processes. The population within each hexagon is based on detailed UK census information, together with more up-to-date commercial information that tracks local changes in housing and occupants' socio-demographics. For each hexagon we therefore are able to create a synthetic population from a known age, gender and employment-status distribution. Aggregating this information generates the national population; therefore for each individual i in England, Wales and Scotland we know their socio-demographic status, ϕ_i, and the hexagon that contains their home, H_i.

Movement is based upon a spatial interaction model, where hexagons have an associated attractiveness for each activity type, k [1,2]. Taking a single representative example will illustrate the processes involved; we will consider individual i and the activity type of food shopping. Given that we know that individual i lives in hexagon H_i and their age, gender and employment-status (as captured by ϕ_i), this provides valuable information on their full socio-demographic status (that we will term $\Phi_i(\phi_i,H_i)$) which in turn informs about spending potential, retail preferences and weekly activity pattern based on amalgamated market-research information. Given the full socio-demographic status (Φ_i) there will be known preferences, $P_s(\Phi_i)$, for a particular shop or supermarket, s, that will depend upon the brand and size of the shop. Locations and sizes of shops (both food and non-food) are commercially available. A cost of travelling to the shop, $c(s,H_i)$ is quantified by estimating travel routes. The number of people of socio-demographic status Φ_i who travel to shop s on day part d, $N_s^d(\Phi_i)$, is estimated by:

$$N_s^d(\Phi_i) = P_s(\Phi_i) \times f(c(s,H_i)) \times O^d(\Phi_i) \times W(\Phi_i) \tag{S7.1}$$

where $O^d(\Phi_i)$ is a measure of the demand on day part d and $W(\Phi_i) = 1 / \sum_s P_s(\Phi_i) f(c(s,H_i))$ is a balancing factor that takes account of the competition and ensures that all demand is allocated to all centres in the region.

The precise functional form of the model is parameterised to match retail patterns, such as those collected through store loyalty cards and electronic point-of-sale records [1,4]. The probability that individual i will visit hexagon x during day part d is then

$$P^d(x \mid \Phi_i) = \sum_{s \in x} N_s^d(\Phi_i) / N_{\Phi_i} \tag{S7.2}$$

where N_{ϕ_i} is the total number of individuals of status Φ_i. An example of the accuracy of the Oxford Retail Consultants' model in predicting the "catchment area", or the area that

contains the residential locations of potential customers, for a particular brand of shop, "ILVA", for the borough of Thurrock, England, is shown in Figure S8.1.

In addition to travel for food shopping activity the database also includes predictions of journeys made for non-food shopping, work, school and higher education activity. Locations, sizes and types of school and work-places are available through public sources such as the England National Pupil Database and the United Kingdom Census, as well as commercial sources. The workplace data provided to Oxford Retail Consultants by Blue Sheep (http://bluesheep.bluegroupinc.com/products/ukbu). Since UK businesses are obliged to register, this will include all businesses, regardless of size, who are operating legally.

While the Oxford Retail Consultants' Great Britain human movement database is clearly a highly detailed representation of human movement patterns based on an amalgamation of various data sources, there are areas of weakness. The main focus of Oxford Retail Consultants has been the retail sector, and therefore movement for social activities has been less developed. Similarly, we may expect fluctuations in travel behaviour over the year that are not captured in the database, either due to temperature and holiday patterns, or particular events (eg sporting fixtures). In addition, the Great Britain human movement database seeks to apply one set of universal rules to the entire population, therefore local knowledge of travel patterns or individual travel-histories are generally likely to be far more detailed and representative of the true situation. Figure S9.1 gives an example of the ability of the database to predict spatial patterns of shopping activity for large subpopulations in the United Kingdom.

Verification of ORC Model Predictions

Predictions from ORC Model

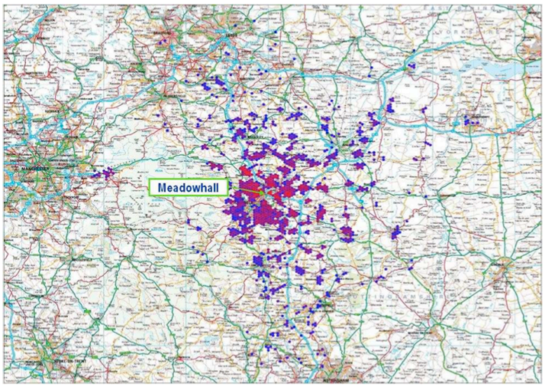

Catchment area of Meadowhall Shopping Centre in Sheffield generated using ORC predictions of sales for one year.

Consumer Survey Data

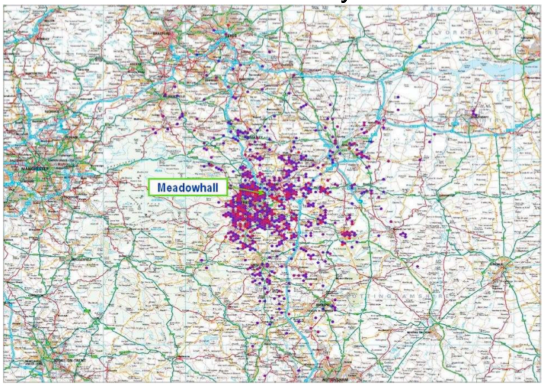

Catchment area of Meadowhall Shopping Centre in Sheffield generated from a sample of 3,500 shoppers. The lower density is due to the relatively small sample size.

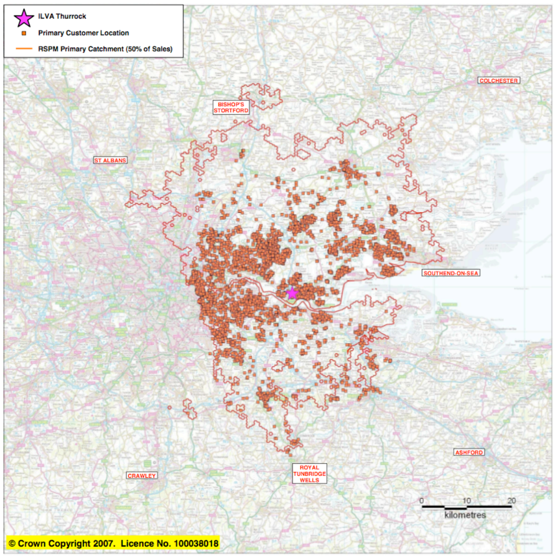

Primary catchment area (region that captures 50% of total spend) for Thurrock. Model results (line), sample data (points)

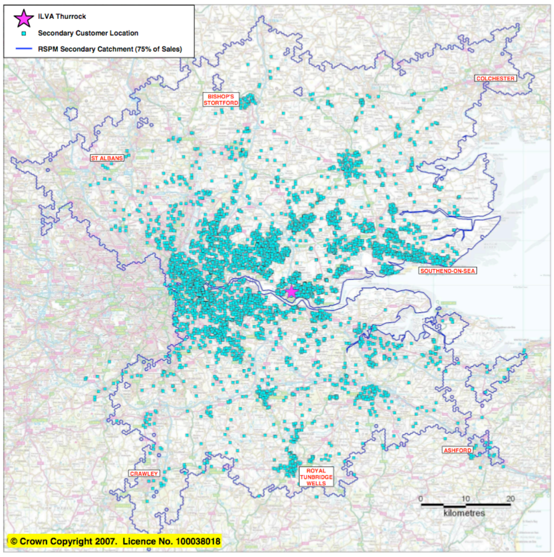

Secondary catchment area (region that captures 75% of total spend) for Thurrock. Model results (line), sample data (points)

Figure S9.1. Verification of the predictions of the Oxford Retail Consultants model for different regions within Great Britain.

References

1. Birkin M, Clarke G, Clarke M (2002) Retail Geography and Intelligent Network Planning. Chichester: John Wiley and Sons, Ltd.
2. Cliquet G (2006) Geomarketing: Methods and Strategies in Spatial Marketing. London: ISTE.
3. Smith G, Arnot C, Fairburn J, Walker G (2005) A national population data base for major accident hazard modelling. Sudbury, Suffolk, UK: HSE Books.
4. Miller CC Cellphone in New Role: Loyalty Card. The New York Times.
